# Vital Signs: Repeat Births Among Teens — United States, 2007–2010

**Published:** 2013-04-05

**Authors:** Lorrie Gavin, Lee Warner, Mary Elizabeth O’Neil, Linh M. Duong, Cassondra Marshall, Philip A. Hastings, Ayanna T. Harrison, Wanda Barfield

**Affiliations:** Division of Reproductive Health, National Center for Chronic Disease Prevention and Health Promotion, CDC

## Abstract

**Background:**

Teen childbearing has potential negative health, economic, and social consequences for mother and child. Repeat teen childbearing further constrains the mother’s education and employment possibilities. Rates of preterm and low birth weight are higher in teens with a repeat birth, compared with first births.

**Methods:**

To assess patterns of repeat childbearing and postpartum contraceptive use among teens, CDC analyzed natality data from the National Vital Statistics System (NVSS) and the Pregnancy Risk Assessment Monitoring System (PRAMS) from 2007–2010.

**Results:**

Based on 2010 NVSS data from all 50 states and the District of Columbia, of more than 367,000 births to teens aged 15–19 years, 18.3% were repeat births. The percentage of teen births that represented repeat births decreased by 6.2% between 2007 and 2010. Disparities in repeat teen births exist by race/ethnicity, with the highest percentages found among American Indian/Alaska Natives (21.6%), Hispanics (20.9%), and non-Hispanic blacks (20.4%) and lowest among non-Hispanic whites (14.8%). Wide geographic disparities in the percentage of teen births that were repeat births also exist, ranging from 22% in Texas to 10% in New Hampshire. PRAMS data from 16 reporting areas (15 states and New York City) indicate that 91.2% of teen mothers used a contraceptive method 2–6 months after giving birth, but only 22.4% of teen mothers used the most effective methods. Teens with a previous live birth were significantly more likely to use the most effective methods postpartum compared with those with no prior live birth (29.6% versus 20.9%, respectively). Non-Hispanic white and Hispanic teens were significantly more likely to use the most effective methods than non-Hispanic black teens (24.6% and 27.9% versus 14.3%, respectively). The percentage of teens reporting postpartum use of the most effective methods varied greatly geographically across the PRAMS reporting areas, ranging from 50.3% in Colorado to 7.2% in New York State.

**Conclusions:**

Although the prevalence of repeat teen birth has declined in recent years, nearly one in five teen births is a repeat birth. Large disparities exist in repeat teen births and use of the most effective contraceptive methods postpartum, which was reported by fewer than one out of four teen mothers.

**Implications for Public Health Practice:**

Evidence-based approaches are needed to reduce repeat teen childbearing. These include linking pregnant and parenting teens to home visiting and similar programs that address a broad range of needs, and offering postpartum contraception to teens, including long-acting methods of reversible contraception.

## Introduction

Although teen birth rates have been declining for the last two decades, in 2010, more than 367,000 teens aged 15–19 years gave birth (1). Teen childbearing has potential negative health, economic, and social consequences for mothers and their children (1,2), and each year teen childbearing costs the United States approximately $11 billion (3). In response, the U.S. government has set a *Healthy People 2020* objective for reducing teen pregnancy rates (4).

Repeat teen birth (defined here as having two or more pregnancies resulting in a live birth before age 20 years) poses greater challenges because additional births might further constrain the mother’s ability to attend school and obtain job experience (5). Closely spaced births also have health consequences for the infant (6). For example, 17.0% of infants who were second teen births were born preterm in 2010, compared with 12.6% for first births; 11% of second teen births were low birth weight, compared with 9% of first births (1,7).

Given that most pregnant teens come into contact with the health-care system while receiving prenatal care (8), opportunities exist to help them prevent subsequent pregnancies in their teen years. The American Academy of Pediatrics and the American College of Obstetricians and Gynecologists recommend counseling women about birth spacing and contraceptive use during pregnancy (9,10). Research among teen mothers has shown that prenatal counseling is associated with an increased likelihood of using contraception and of using more effective methods (11), and that use of effective methods is associated with reduced rates of repeat teen pregnancy (12). Home visiting and similar programs that provide broad-based support to pregnant and parenting teens have been shown to reduce repeat teen pregnancy (13).

To assess patterns of repeat teen childbearing and postpartum contraceptive use, CDC analyzed data from the natality files of the National Vital Statistics System (NVSS), and the Pregnancy Risk Assessment Monitoring System (PRAMS). Specific research questions included the following:

1. What number and percentage of teen births are repeat births?
2. What are patterns of repeat teen births by race/ethnicity, by state, and over time?
3. What are current patterns of postpartum contraceptive use among teen mothers, by sociodemographic characteristics and by state?

## Methods

### Vital Statistics/Birth Data

U.S. natality files are compiled annually by CDC’s National Center for Health Statistics and include demographic information such as maternal age, race, and Hispanic origin for all births in the United States in all states and the District of Columbia. This report includes national and state-specific data for 2007–2010 (7). For the analyses, births to females aged 15–19 years for which information about the number of prior pregnancies ending in a live birth was not available (less than 1% of births in 2010) were excluded, leaving 364,859 births for these analyses.

### PRAMS

To examine contraceptive methods used by teen mothers postpartum, CDC analyzed data from the Pregnancy Risk Assessment Monitoring System (PRAMS) (14). PRAMS collects state-specific, population-based data on maternal attitudes and experiences before, during, and after pregnancy. In each participating state, a stratified random sample of mothers with a recent live birth is selected from the birth files and, using a standardized protocol, women are surveyed by mail 2–6 months after the birth of their child, with telephone follow-up as needed. PRAMS data are weighted for sample design, nonresponse, and noncoverage using birth certificate data provided by vital statistics agencies in the participating states, to produce an analysis dataset representative of the state birth population. The analysis in this report included data from 2007–2010 from a subset of 16 reporting areas (15 states* and New York City, representing 28% of all live births) that had PRAMS data necessary to conduct the analysis, and a weighted response rate ≥65%.

All respondents were asked, “Are you or your husband or partner doing anything *now* to keep from getting pregnant?” If the response was no, the mother was asked the reason from a list of response choices, with instructions to “check all that apply.” If the response was yes, respondents were asked to check all applicable responses to the question, “What kind of birth control are you or your husband or partner using *now* to keep from getting pregnant?” Contraceptive methods were categorized by level of effectiveness for pregnancy prevention based on the percentage of females who experience pregnancy during the first year of typical use and coded in a manner consistent with previous analyses of contraceptive use as most effective (<1%), moderately effective (6%–12%), and less effective (≥18%) (15). Only the most effective method listed by the respondent was used in the categorizations. The *most effective* methods included tubal ligation, vasectomy, implant, and intrauterine device; *moderately effective* included oral contraceptive pills, injectable medroxyprogesterone (e.g., Depo-Provera), birth control patch, and vaginal ring; and *less effective* included condom, diaphragm, cervical cap, contraceptive sponge, rhythm method, and withdrawal during typical use. Although the diaphragm has been categorized elsewhere as moderately effective during typical use (15), for this report, that method was categorized as less effective because the PRAMS question combines diaphragm/cap/sponge as a single response option, making it impossible to determine which method was used. However, the eight teens reporting use of a diaphragm also reported use of another contraceptive method with a higher level of effectiveness.

Weighted prevalences were calculated using statistical software to account for the complex sampling design and nonresponse. Weighted results were calculated for female teens aged <20 years whose pregnancy resulted in a live birth. The sample included teen mothers who recently had delivered their first child and were at risk for having a second birth, as well as teen mothers who recently had delivered a subsequent child and were at risk for having a third or higher order birth. Analyses examining the typical use effectiveness of contraceptive methods and reasons for nonuse of contraception excluded teen mothers who were not at risk for pregnancy, either because they currently were pregnant or were not sexually active. The prevalence of self-reported contraceptive use postpartum was estimated by select demographic characteristics and reasons for not using contraception were characterized.

## Results

### Vital Statistics

In 2010, among 364,859 births to teens aged 15–19 years, 66,761 (18.3%) represented repeat births. The vast majority (85.7%) of repeat births were for a second child (57,206 of 66,761), but some teens (12.6%) were giving birth to a third child (8,397), and a few births (1.7%) were for a fourth to sixth child (1,158). The percentage of teen births that represented repeat births decreased gradually over the observation period, from 19.5% in 2007 to 18.3% in 2010, for a 6.2% decline over the 4-year period.

The prevalence of repeat teen births varied by race/ethnicity, with the highest prevalence in 2010 among American Indian/Alaska Natives (21.6%), followed by Hispanics (20.9%), non-Hispanic blacks (20.4%), Asian or Pacific Islanders (17.6%), and non-Hispanic whites (14.8%). The prevalence of repeat teen births also varied by state (Figure). The highest prevalence (22%) was found in Texas, while the lowest prevalence (10%) was found in New Hampshire. In eight southern and western states (Arizona, Arkansas, Georgia, Louisiana, Mississippi, Nevada, Oklahoma, and Texas,), ≥20% of all teen births to females aged 15–19 years were repeat births. Conversely, in seven mostly northeastern states (Connecticut, Maine, Massachusetts, New Hampshire, New York, Vermont, and Wyoming) <15% of all teen births were repeat births.

### PRAMS

Among postpartum teen mothers from the participating PRAMS reporting areas, 8.0% were not sexually active (95% confidence interval [CI] = 6.9%–9.2%), 1.3% were pregnant (CI = 0.9%–1.9%), and 90.7% were sexually active (CI = 89.4%–91.9%). Teen mothers with a repeat birth were as likely as teen mothers with a first birth to report the birth was unintended (72.7% [CI = 68.0%–77.0%] versus 72.6% [CI = 70.3%–74.7%], respectively), and to report using contraception in the prepregnancy period before the birth (48.8% [CI = 43.1%–54.6%] versus 45.6% [CI = 42.9%–48.3%], respectively).

Of teen mothers who were sexually active, 91.2% reported using postpartum contraception after the most recent birth. Among sexually active teen mothers, 22.4% used the most effective birth control methods, 54.2% used moderately effective methods, 14.5% used less effective methods, and 8.8% used no method (Table 1). Teens with a previous live birth were significantly more likely to use the most effective methods compared with those with no prior live birth (29.6% versus 20.9%) (Table 1). Non-Hispanic white and Hispanic teens were significantly more likely to use the most effective methods than Non-Hispanic black teens (24.6% and 27.9% versus 14.3%, respectively (Table 1). Usage also differed somewhat by age, with teens aged ≤17 years more likely than teens aged 18–19 years to use moderately effective methods (60.4% versus 51.4%), and less likely to use the less effective methods (11.2% versus 16.0%); however, there were no significant differences in use of the most effective methods. Use of the most effective methods did not differ significantly between married and other teens; however, married teens were less likely to use moderately effective methods (41.6% versus 56.4%); and more likely to use no method (15.9% versus 7.6%), which could reflect pregnancy intendedness among married teens.

Postpartum use of effective contraception among teen mothers also varied markedly by location (Table 1). Of the 16 PRAMS reporting areas in the sample, Colorado had the highest percentage of teen mothers reporting use of the most effective birth control methods postpartum (50.3%), compared with New York State (excluding New York City), which had the lowest percentage (7.2%) (Table 1). New York City reported the highest percentage of no birth control use postpartum (19.2%), while South Carolina reported the lowest percentage (4.1%) (Table 1).

Among teen mothers who used contraception postpartum, more than one out of every five respondents reported using long-acting reversible contraception (LARC), with 18.2% reporting intrauterine device use and 3.3% reporting implant use (Table 2). Use of oral contraceptive pills and the shot (Depo Provera) was reported by 29.2% and 21.0% of teen mothers postpartum, respectively. Among respondents, 12.3% reported using condoms as their method of preventing pregnancy.

Among the 9% of sexually active teen mothers who did not use birth control after their most recent birth, the most frequently cited reasons for nonuse included not wanting to use birth control (36.0% [CI = 28.7%–44.0%]), husband/partner objections (21.7% [CI = 15.9%–28.9%]), not being able to pay for birth control (20.0% [CI = 14.5%–26.8%]), and wanting to get pregnant (17.6% [CI = 12.1%–24.9%]).

## Conclusions and Comment

This report documents that nearly one in five teen births in 2010 was a repeat birth. The percentage of teen births that were repeat births has decreased 6.2%, from 19.5% in 2007 to 18.3% in 2010. The prevalence of repeat teen births varied by race/ethnicity, and mirrored racial/ethnic disparities in the overall teen birth rates with Hispanics, non-Hispanic blacks, and American Indian/Alaska Natives experiencing the highest rates (1).

This report also examined postpartum contraceptive use, a proximal determinant of the risk for a repeat teen birth. Overall, 91% of teens with a recent live birth reported using contraception postpartum; this represents a substantial increase from the 45%–50% of teens with a recent live birth who reported using contraception in the prepregnancy period. This percentage also is similar to the percentage of all sexually active female teens (85.6%) who reported use of a method of birth control at last sex (16). More than three quarters of sexually active teen mothers used one of the most or moderately effective contraceptive methods postpartum, and teen mothers were more likely to use LARC than all sexually active teens (21.5% versus 4.5%) (17). Of note, a previous report of PRAMS data from seven states in 2006–2008 showed only 12% of teen mothers were using LARC (11). The more recent data from 16 PRAMS reporting areas suggest that an increasing percentage of teen mothers are actively attempting to prevent another pregnancy in the postpartum period through use of the most effective methods of contraception.

Another way to reduce repeat teen pregnancy is to engage pregnant and parenting teens in programs that are effective in reducing repeat teen births. Several studies have shown that home visiting can help reduce repeat teen pregnancy (5,13). The U.S. Department of Health and Human Services (HHS) Maternal and Child Health Bureau helps states and local agencies deliver evidence-based home visiting programs.† The HHS Office of Adolescent Health’s Pregnancy Assistance Fund (PAF) Resource and Training Center also provides information and tools for use by those working with pregnant and parenting teens.§

Efforts to support pregnant and parenting teens should include counseling about birth spacing and contraception and, among women wishing to delay or avoid future pregnancies, the importance of sustaining contraceptive use over time, in accordance with recommendations from professional organizations such as the American Academy of Pediatrics and the American College of Obstetricians and Gynecologists (9,10). LARC methods are safe and effective for most teens (18). Given that teens are at a high risk for inconsistent use of methods that are user-dependent (e.g., condoms and oral contraceptive pills), LARC methods might be a suitable option because they are user independent and require no effort after insertion (19). However, teens face a number of barriers to LARC use, including cost, limited availability, lack of provider acceptance for this practice in teens, and teen lack of awareness of these methods (20). Counseling about birth spacing and contraception during pregnancy and offering LARC in the immediate postpartum period while in the hospital after delivery are examples of how to successfully facilitate contraceptive access for teen mothers. In addition, consistent and correct condom use should be encouraged to prevent sexually transmitted infections, including human immunodeficiency virus infection.

The wide geographic variation in use of the most effective contraceptive methods among the PRAMS reporting areas included in this analysis could be explained by a number of factors, such as environmental support for contraception, that should be explored further. Research should attempt to identify how some states successfully overcame barriers to use of the most effective method postpartum. Moreover, further research should investigate reasons for lower rates of use of the most effective contraceptive methods among non-Hispanic black teens.

The findings in this report are subject to at least five limitations. First, respondents from PRAMS were interviewed in the period shortly after giving birth; later follow-up is needed to better understand longer-term use of postpartum contraception and determinants of repeat teen childbearing. Second, the PRAMS data do not include information about the consistency and correctness of contraceptive use, which are particularly important determinants of the effectiveness of user-dependent methods of contraception such as condoms and pills. Third, because only 16 PRAMS reporting areas were included in this analysis, results might not be generalizable to other states. Fourth, the years covered by the analysis span from 2007 to 2010, and averaging estimates over these 4 years could mask temporal trends in contraceptive use given continued declines observed in teen birth rates. States with data only for 2007 and 2008 also might have experienced substantial improvements in later years. Finally, the data sources used for these analyses permitted examination only of repeat births among teens rather than repeat pregnancies; because miscarriages, stillbirths, and abortions were not included, the prevalence of repeat pregnancy likely is higher than repeat births.

**Key Points**

- Having more than one child during the teen years might pose greater challenges than having one child. Rates of preterm and low birth weight are higher among repeat teen births than among first births.
- Nearly one in five teen births (nearly 67,000 in 2010) is a repeat birth.
- Many teens are taking actions to prevent repeat pregnancies and births. Most (91%) sexually active teen mothers are using contraception in the postpartum period, but only 22% are using the most effective methods.
- Postpartum contraceptive use varies by the number of previous births, race/ethnicity, and geographic location. The geographic variation suggests that barriers might exist to accessing contraception, including the most effective methods of reversible contraception.
- What can be done to help reduce repeat teen births?
  – Work with teens during pregnancy at prenatal visits.
  – Provide broader support and link pregnant and parenting teens to sources of educational, economic, and social support that should continue after the child is born.
  – Counsel teens about abstinence and contraception as a way to prevent pregnancy, and promote condom use to prevent pregnancy and sexually transmitted infections, including human immunodeficiency virus.
  – Encourage providers to offer postpartum contraception to teens.
- Information for those working with pregnant and parenting teens is available from the U.S. Department of Health and Human Services’ Office of Adolescent Health’s Pregnancy Assistance Fund Resource and Training Center at http://www.hhs.gov/ash/oah/oah-initiatives/paf.
- The Maternal and Child Health Bureau of the Health Resources and Services Administration is helping state and local agencies deliver evidence-based home visiting programs. Additional information is available at http://mchb.hrsa.gov/programs/homevisiting.
- Additional information is available at http://www.cdc.gov/vitalsigns.

The findings in this report suggest that many teen mothers are taking steps in the postpartum period to prevent repeat pregnancy. Previous research has shown that these efforts can be supported by linking pregnant and parenting teens to home visiting programs and other sources of support, as well as health care that includes counseling about and provision of contraception (5,9,16).

## Figures and Tables

**FIGURE:**
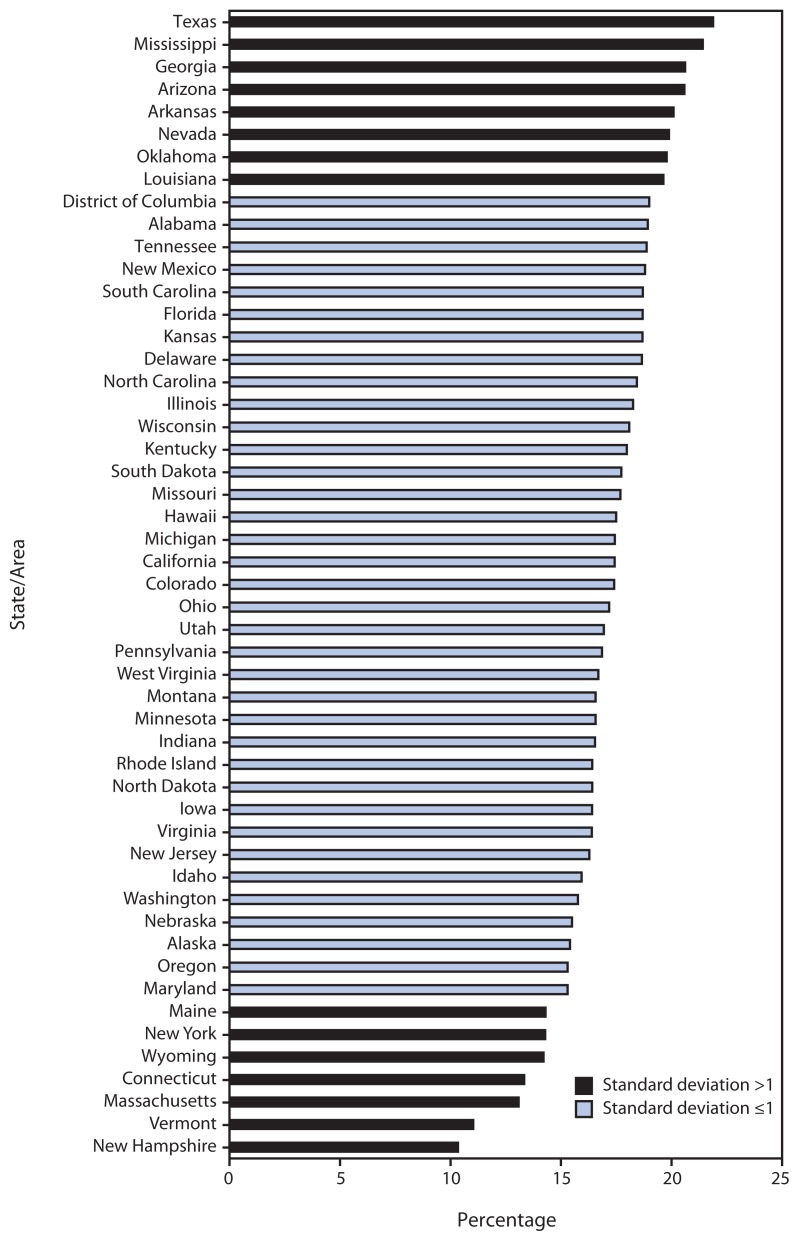
Percentage of births* among females aged 15–19 years that were repeat births, by state/area — United States, 2010 * Excludes births for which the birth order was not known.

**TABLE 1:** Postpartum contraceptive use among nonpregnant, sexually active females aged <20 years who delivered live infants, by selected characteristics — 15 states and New York City, Pregnancy Risk Assessment Monitoring System (PRAMS), 2007–2010

Characteristic	No. in sample*	%†	Most effective§		Moderately effective¶		Less effective**		No method	
			
			%	(95% CI)	%	(95% CI)	%	(95% CI)	%	(95% CI)
**Total**	**5,708**	**100.0**	**22.4**	**(20.6–24.3)**	**54.2**	**(51.9–56.5)**	**14.5**	**(13.0–16.2)**	**8.8**	**(7.6–10.3)**
**Previous live birth**										
Yes	1,026	17.3	29.6	(24.9–34.8)	44.5	(39.2–50.0)	16.6	(13.0–20.9)	9.3	(6.5–13.3)
No	4,656	82.7	20.9	(19.0–23.0)	56.1	(53.6–58.6)	14.2	(12.5–16.1)	8.8	(7.4–10.4)
**Race/Ethnicity**										
White, non-Hispanic	2,673	56.9	24.6	(21.9–27.4)	53.1	(49.8–56.3)	13.0	(11.0–15.4)	9.3	(7.6–11.4)
Black, non-Hispanic	1,540	25.3	14.3	(11.7–17.4)	63.7	(59.6–67.7)	15.6	(12.7–19.1)	6.3	(4.4–8.9)
Hispanic	1,009	17.8	27.9	(23.6–32.6)	44.0	(38.8–49.4)	17.5	(13.8–21.9)	10.6	(7.7–14.4)
**Age (yrs)**										
≤17	1,795	31.9	20.7	(17.7–24.2)	60.4	(56.3–64.3)	11.2	(9.0–14.0)	7.7	(5.7–10.2)
18–19	3,913	68.1	23.2	(21.0–25.6)	51.4	(48.6–54.1)	16.0	(14.1–18.2)	9.4	(7.9–11.2)
**Marital status**										
Married	873	14.5	26.7	(21.9–32.1)	41.6	(36.1–47.4)	15.8	(12.4–19.9)	15.9	(12.0–20.9)
Other	4,830	85.5	21.7	(19.7–23.8)	56.4	(53.9–58.8)	14.3	(12.6–16.2)	7.6	(6.4–9.1)
**State/City** ††										
Arkansas	829	8.2	17.7	(14.3–21.7)	53.8	(49.0–58.6)	16.4	(13.2–20.3)	12.0	(9.2–15.6)
Colorado (2009, 2010)	295	4.6	50.3	(42.0–58.6)	35.2	(27.9–43.2)	9.0	(5.4–14.6)	5.5	(2.7–10.8)
Michigan	678	19.9	20.2	(16.6–24.4)	59.5	(54.7–64.1)	13.2	(10.4–16.7)	7.1	(5.0–10.1)
Missouri (2007)	126	3.3	15.5	(9.2–25.0)	67.0	(57.0–75.6)	11.1	(6.9–17.4)	6.4	(3.4–11.8)
Mississippi (2008)	235	2.7	15.8	(10.8–22.6)	68.2	(60.2–75.2)	6.9	(3.7–12.5)	9.1	(5.4–14.9)
North Carolina (2007, 2008)	303	11.8	24.9	(19.4–31.5)	48.2	(41.3–55.2)	17.4	(12.7–23.4)	9.4	(6.0–14.5)
Nebraska	514	2.9	27.8	(23.2–32.8)	47.7	(42.2–53.2)	15.2	(11.8–19.4)	9.3	(6.4–13.3)
New York (2007, 2008)§§	112	7.0	7.2	(3.2–15.6)	70.2	(57.9–80.2)	14.1	(7.3–25.5)	8.4	(3.7–17.9)
New York City (2007)	81	3.4	11.9	(5.1–25.4)	43.9	(31.0–57.6)	25.0	(14.9–38.9)	19.2	(10.6–32.3)
Ohio (2009, 2010)	249	11.0	23.5	(16.6–32.1)	55.1	(46.1–63.9)	14.5	(9.4–21.7)	6.9	(3.2–14.2)
Oregon	554	5.9	33.8	(27.6–40.6)	43.5	(36.8–50.3)	15.1	(10.9–20.6)	7.6	(5.0–11.4)
Rhode Island	406	1.7	36.4	(31.0–42.2)	44.4	(38.7–50.3)	10.4	(7.4–14.5)	8.8	(6.0–12.6)
South Carolina (2007)	184	3.1	15.5	(8.5–26.7)	64.1	(51.4–75.1)	16.2	(8.9–27.7)	4.1	(1.3–11.9)
Tennessee (2008, 2009)	177	9.8	20.7	(13.9–29.5)	54.0	(44.5–63.3)	13.0	(7.9–20.7)	12.3	(7.2–20.2)
Utah (2009, 2010)	249	2.8	40.3	(33.8–47.2)	34.0	(27.7–41.0)	18.8	(13.9–24.9)	6.9	(4.3–10.9)
West Virginia (2007, 2008)	716	1.9	11.2	(8.5–14.6)	63.1	(58.4–67.6)	13.8	(10.9–17.4)	11.8	(9.0–15.4)

**Abbreviation:** CI = confidence interval.

* Unweighted sample totals from 5,708 females age <20 years responding that they were not pregnant and were sexually active. If more than one method of contraception was reported, only the method with the highest effectiveness during typical use was included.

† Percentages based on weighted data; totals might not sum to 100% because of rounding or missing data for some categories.

§ Includes tubal ligation, vasectomy, implant, and intrauterine device. Effectiveness determined by the percentage of females who experience pregnancy during first year of typical use; categorized as most effective (<1%), moderately effective (6%–12%), and less effective (≥18%).

¶ Includes oral contraceptive pills, injectable medroxyprogesterone (e.g., Depo-Provera, also known as the birth control shot), birth control patch, and vaginal ring.

** Includes condom, diaphragm, cervical cap, contraceptive sponge, rhythm method, and withdrawal.

†† The following sites did not have complete data for all years of 2007–2010: Colorado, Missouri, Mississippi, North Carolina, New York, Ohio, South Carolina, Tennessee, Utah, West Virginia, and New York City; the year(s) in parentheses indicates for which year(s) data were available for these states. New York City did have complete data in 2010, but was excluded from this year’s analysis because the list of contraceptive method types was modified and did not correspond to the other states and years.

§§ Excluding New York City.

**TABLE 2:** Postpartum contraceptive* use among nonpregnant, sexually active females aged <20 years who delivered live infants — 15 states and New York City,† Pregnancy Risk Assessment Monitoring System (PRAMS), 2007–2010

Characteristic	No. in sample§	%¶	(95% CI)
**Total**	**5,708**	**100.0**	**—**
Any use	5,179	91.2	(89.7–92.4)
**Most effective**			
Tubal ligation	26	0.3	(0.1–0.6)
Vasectomy	20	0.6	(0.3–1.0)
Implant	175	3.3	(2.6–4.2)
Intrauterine device	1,058	18.2	(16.6–20.0)
**Moderately effective**			
Pill	1,615	29.2	(27.1–31.3)
Shot once a month or shot once every 3 months (e.g., Depo-Provera)	1,173	21.0	(19.2–23.0)
Patch	114	1.8	(1.3–2.5)
Ring	148	2.3	(1.7–2.9)
**Less effective**			
Condom	747	12.3	(10.9–13.8)
Diaphragm/Cap/Sponge	0	—	—
Rhythm	22	0.5	(0.2–1.0)
Withdrawal	81	1.8	(1.2–2.6)
No method	529	8.8	(7.6–10.3)

**Abbreviation:** CI = confidence interval.

* Effectiveness determined by the percentage of females who experience pregnancy during the first year of typical use; categorized as most effective (<1%), moderately effective (6%–12%), and less effective (≥18%). If more than one method was reported; only the most effective method was included.

† Sites included Arkansas, Colorado, Michigan, Missouri, Mississippi, North Carolina, Nebraska, New York (excluding New York City), Ohio, Oregon, Rhode Island, South Carolina, Tennessee, Utah, West Virginia, and New York City.

§ Unweighted sample totals from 5,708 females aged <20 years responding that they were not pregnant and were sexually active. If more than one method of contraception was reported, only the method with the highest effectiveness during typical use was included.

¶ Percentages based on weighted data; totals might not sum to 100% because of rounding or missing data for some categories.

## Footnotes

* States included Arkansas, Colorado, Michigan, Missouri, Mississippi, North Carolina, Nebraska, New York (excluding New York City), Ohio, Oregon, Rhode Island, South Carolina, Tennessee, Utah, and West Virginia.

† Additional information about home visiting is available at http://mchb.hrsa.gov/programs/homevisiting.

§ The HHS Office of Adolescent Health’s Pregnancy Assistance Fund Resource and Training Center provides information to those working with pregnant and parenting teens. Additional information is available at http://www.hhs.gov/ash/oah/oah-initiatives/paf.
